# Diagnostic Accuracy of the Eating Assessment Tool-10 (EAT-10) in Screening Dysphagia: A Systematic Review and Meta-Analysis

**DOI:** 10.1007/s00455-022-10486-6

**Published:** 2022-07-18

**Authors:** Ping-ping Zhang, Ying Yuan, De-zhi Lu, Ting-ting Li, Hui Zhang, Hong-ying Wang, Xiao-wen Wang

**Affiliations:** 1 School of Rehabilitation Medicine, Weifang Medical University, 7166 Baotong West Street, Weifang, Shandong China; 2 School of Medical, Shanghai University, 99 Shangda Road, Shanghai, China; 3 Rehabilitation Department, Affiliated Hospital of Weifang Medical College, 518 Fuyuan Street, Weifang, Shandong China

**Keywords:** Dysphagia, Sensitivity, Specificity, Screening, Meta-analysis

## Abstract

The Eating Assessment Tool-10 (EAT-10) is used worldwide to screen people quickly and easily at high risk for swallowing disorders. However, the best EAT-10 cutoff value is still controversial. In this systematic review and meta-analysis, we estimated and compared the diagnostic accuracy of EAT-10 cutoff values of 2 and 3 for screening dysphagia. We searched the PubMed, Web of Science, EMBASE, Cochrane Library, CNKI, WANFANG, and VIP databases from May 2008 to March 2022. The meta-analysis included 7 studies involving 1064 subjects from 7 different countries. Two studies were classified as high quality and five studies as medium quality. With an EAT-10 cutoff value of 2, using flexible endoscopic evaluation of swallowing or video fluoroscopic swallowing study as the gold standard, the pooled sensitivity, specificity, positive and negative likelihood ratios, and diagnostic odds ratio were 0.89 (95% confidence interval [CI] 0.82–0.93), 0.59 (95% CI 0.39–0.77), 2.17 (95% CI 1.38–3.42), 0.19 (95% CI 0.13–0.29), and 11.49 (95% CI 5.86–22.53), respectively. When a cutoff of 3 was used, these values were 0.85 (95% CI 0.68–0.94), 0.82 (95% CI 0.65–0.92), 4.84 (95% CI 1.72–13.50), 0.18 (95% CI 0.07–0.46), and 26.24 (95% CI 5.06–135.95), respectively. Using EAT-10 cutoff values of 2 and 3, the areas under the curve were 0.873 (95% CI 0.82–0.93) and 0.903 (95% CI 0.88–0.93), respectively, showing good diagnostic performance. EAT-10 can be used as a preliminary screening tool for dysphagia. However, a cutoff of 3 is recommended for EAT-10 due to better diagnostic accuracy.

## Introduction

Dysphagia refers to a sensation or sign of an underlying health problem in which food or liquid has difficulty entering the stomach [1–3]. Aging is an independent risk factor for swallowing disorders, and the prevalence of dysphagia among adults over 60 years of age living independently is 11.4–33.7% [4, 5]. Addition, cerebrovascular and neurodegenerative diseases cause a broad spectrum of neurological disorders and lead to difficulty in swallowing [6]. The prevalence of dysphagia in stroke is 29–64% [7, 8], 50–75% in Alzheimer’s disease [9], and can be as high as 82% in Parkinson’s [10]. Head and neck cancer, trauma, gastroesophageal reflux disease, and primary esophageal abnormalities are also common causes of dysphagia [11]. The health and economic impact of dysphagia on patients are enormous, and common complications include dehydration, malnutrition, and aspiration pneumonia. Patients with dysphagia have significantly longer hospital stays, are four times more likely to be readmitted within a month, and have a 13-fold higher mortality rate during hospitalization [12–14]. Bonilha et al. [15] followed patients with dysphagia for 1 year after controlling for influencing factors and found that annual medical costs for these patients were 4510 USD higher than for patients without dysphagia. At present, the video fluoroscopic swallowing study (VFSS) and flexible endoscopic evaluation of swallowing (FEES) are considered the gold standards for the diagnosis of dysphagia, both of which are imaging methods with high consistency in penetration, aspiration, and pharyngeal residue assessments [16, 17]. Eleven studies [18–27] have reported that patients are exposed to x-ray radiation for 2.5 to 18 min during VFSS examination, and Bonilha et al. [28] assessed 612 patients and identified an average exposure time of 2.9 min. FEES is only for local observation, and it cannot be used to observe pharyngeal contraction, laryngeal activity, or cricopharyngeal muscle opening during swallowing [29]. At the same time, owing to individual economic and medical development levels and health policies, it is difficult to evaluate every patient at risk of swallowing disorders. These individuals should first be screened using a questionnaire and those with suspected dysphagia can be further evaluated using imaging evaluation. Early screening can identify people at risk for swallowing disorders, and appropriate feeding strategies and treatment can also improve patient prognosis and reduce the incidence of aspiration pneumonia [30]. Although several screening tools for dysphagia have been developed, few are actually widely used in clinical practice. Sensitivity and specificity are important indicators of the performance of screening tools, but some screening tools have low diagnostic accuracy. There are little data on sensitivity and specificity for the Sydney Swallow Questionnaire (SSQ), the Dysphagia Risk Assessment for Community-Dwelling Older Adults (DRACE), and the Ohkuma questionnaires [31, 32]; the Modified Mann Assessment of Swallowing Ability (MMASA), Munich Dysphagia Test-Parkinson’s Disease (MDT-PD), and the Mayo Dysphagia Questionnaire (MDQ) are tedious and time-consuming; and the Dysphagia in Multiple Sclerosis (DYMUS), M.D. Anderson Dysphagia Inventory (MDADI), and MetroHealth Dysphagia Screen (MDS) questionnaires are only applicable to specific patient groups [11, 32, 33].

The Eating Assessment Tool-10 (EAT-10) is a dysphagia screening tool developed in 2008 by Belafsky et al. [11] to identify people at high risk of swallowing disorders. EAT-10 is currently being used in clinical settings worldwide, has been translated into Chinese [34], Spanish [35], Swedish [36], Italian [37], Brazilian Portuguese [38], European Portuguese [39], Hebrew [40], Greek [41], French [42], and other languages. It is a 10-item self-assessment scale that patients can complete in a short period of time. Each item corresponds to 5 levels of difficulty from “no problem” to “serious problem,” with a total score of 0 to 40. EAT-10 has good internal consistency and intraclass correlation coefficients (ICCs) [11, 34, 36, 37, 39, 40, 42–47]. It has been proven to be useful in screening dysphagia in the oropharyngeal and esophageal phases [11, 44] and for swallowing disorders in a healthy population. There is a good correlation between the EAT-10 score and pharyngeal residual, penetration, and aspiration by FEES and VFSS [40, 48]. All of a patient’s symptoms can be assessed in 3 min [41, 44]. EAT-10 is low-risk for general practitioners, nurses, and other healthcare providers and requires no specialized training. However, there are differences in the reported sensitivity and specificity of EAT-10, and the best cutoff value remains controversial [44, 47, 49]. This study investigated the diagnostic accuracy of EAT-10 screening for dysphagia by meta-analysis, when FEES or VFSS were used as the gold standard and identified the optimal EAT-10 cutoff value to better guide clinical application.

## Methods

The protocol was registered prospectively on PROSPERO (CRD42022300293).

### Study Search

We searched PubMed, Web of Science, EMBASE, the Cochrane Library, CNKI, WANFANG, and VIP from May 2008 (when EAT-10 was developed) to March 2022. The search terms were as follows: (“dysphagia” OR “deglutition disorders” OR “swallowing”) AND (“EAT-10” OR “Eating Assessment Tool”). We also manually searched the references for the selected articles to obtain other eligible studies.

### Study Selection

The inclusion criteria were as follows: age ≥ 18 years, clear consciousness, ability to understand and cooperate to complete the questionnaire, screened by EAT-10 with a cutoff value of 2 or 3, diagnostic gold standard of FEES or VFSS, dysphagia grade of Penetration-Aspiration Scale (PAS) ≥ 2, and the results of the study can be used to directly or indirectly obtain true-positive (TP), false-positive (FP), true-negative (TN), and false-negative (FN) rates.

The exclusion criteria were as follows: conference papers, case reports, letters, and reviews; significant missing data; data in the four-compartment table were not available; the authors did not reply to contact; the quality of the study was poor; the quality evaluation grade was C; or errors in statistical methods.

### Data Extraction

Two trained researchers independently screened, evaluated, and extracted the literature and then cross-checked the literature. In cases of disagreement, the third researcher decided on whether to include the study. Researchers completed preliminary screening of the literature by reading the title, abstract, and keywords of the literature. After reading full texts and determining the literature to be included, the following data were extracted: first author, country, year of publication, study design, number of patients, study population, reference standard, cutoff value, and TP, FP, TN, and FN rates. For incomplete studies, the original author was contacted to the greatest extent possible.

### Quality Assessment

The Quality Assessment of Diagnostic Accuracy Studies (QUADAS-2) was used to assess the quality of the included literature by two researchers and a third reassessed studies in cases of disagreement. There were 4 studies with 10 different evaluation results. The risk of bias was evaluated in four parts: patient selection, index test, reference standard, and flow and timing. The QUADAS-2 scale has a total of 14 items, each with three evaluation standards: “yes” indicates that the standard is met, “no” indicates that the study does not meet the standard or it is not mentioned, and “unclear” indicates that relevant information cannot be obtained from the literature. Finally, the literature was assessed as high, medium, or low quality.

### Data Analysis

Meta-Disk 1.4 [50] was used to test heterogeneity. The diagnostic threshold effect test was performed using Spearman’s correlation coefficient; if *P* > 0.05, it was considered that there was no threshold effect, and each date could be combined. The Cochran–*Q* test for the diagnostic odds ratio (DOR) was used to detect heterogeneity caused by non-threshold effects. *I*^2^ > 50% suggested considerable heterogeneity. The area under the curve (AUC) and Q index were calculated to evaluate the diagnostic accuracy of EAT-10 at cutoff values of 2 and 3. RevMan 5.3 [51] was used to draw summary receiver operating characteristic (SROC) curves. TP, FP, TN, and FN values were input into STATA 16.0 (StataCorp, College Station, TX) to calculate the summary sensitivities, specificities, positive and negative likelihood ratios (PLRs and NLRs), and summary DORs with 95% confidence intervals (CIs).

## Results

### Study Selection

**Fig. 1:**
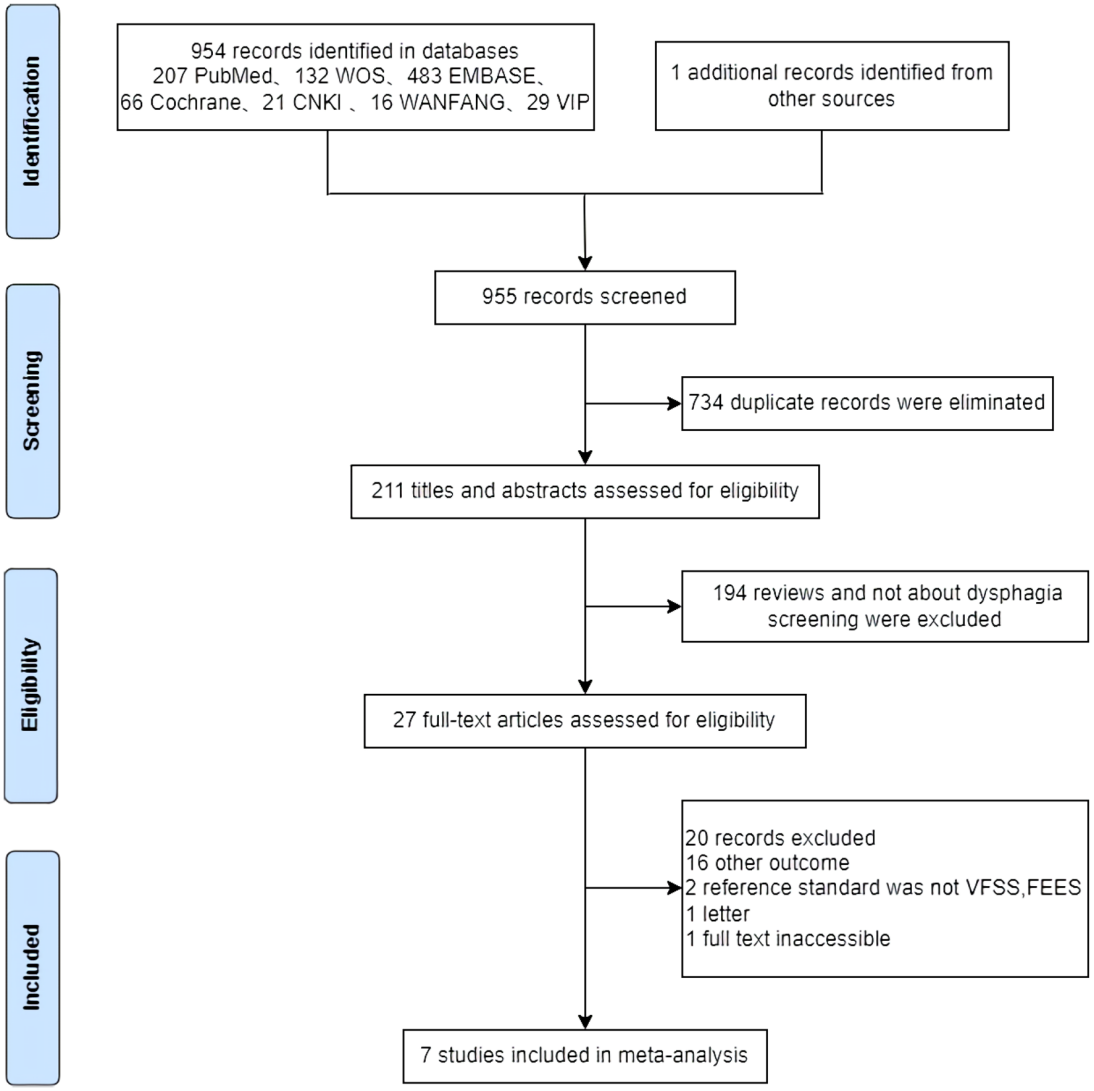
Flowchart of the study selection

A total of 955 related studies were preliminarily identified and 734 duplicate studies were excluded. The researchers read the titles and abstracts for preliminary screening of the literature, carefully read the full text, and evaluated the literature quality. Finally, seven studies were included. A literature screening flowchart is shown in Fig. 1.

### Study Characteristics

The meta-analysis included 1064 subjects from 7 countries, including healthy people and people at high risk for dysphagia, such as those with PD [52], stroke [34], amyotrophic lateral sclerosis (ALS) [53], aging [49], neurodegenerative diseases [36, 49], demyelinating disease [44], and gastroesophageal reflux disease [44]. Four studies [34, 44, 49, 53] used VFSS and three studies [36, 47, 52] used FEES as the gold standard for reporting the sensitivity and specificity of EAT-10. Cross-sectional studies [34, 36, 44, 52, 53] and case–control studies [47, 49] were included. The sensitivity and specificity with an EAT-10 cutoff of 2 were 81.48–93.58% and 36.36–82.35% and 48.65–98.25% and 52.73–98.25% with a cutoff of 3, respectively. The specific features of the literature are listed in Table 1.

**Table 1:** Characteristics of the included studies

Study	Year	Country	Study design	Sample size	Population	Gold standard	Cutoff value	Sensitivity	Specificity	PPV	NPV
Rofes [39]	2014	Spain	Case–control	131	High risk group for dysphagia + healthy group	VFSS	≥ 2	89.47%	82.35%	97.14%	53.85%
							≥ 3	85.09%	82.35%	97.00%	45.16%
RuMi [23]	2015	China	Cross-sectional	130	Stroke	VFSS	≥ 2	81.48%	68.42%	64.71%	83.87%
							≥ 3	57.35%	90.32%	86.67%	65.88%
Plowman [43]	2016	USA	Cross-sectional	70	Amyotrophic lateral sclerosis	VFSS	≥ 3	88.00%	56.70%	52.40%	89.70%
Giraldo-Cadavid [33]	2016	Colombia	Cross-sectional	133	High risk group for dysphagia + healthy group	VFSS	≥ 2	93.58%	36.36%	67.59%	80.00%
							≥ 3	82.05%	52.73%	71.11%	67.44%
Möller [25]	2016	Swedish	Cross-sectional	253	High risk group for dysphagia + healthy group	FEES	≥ 3	98.25	94.96	94.12	98.51
Schlickewei [42]	2021	Germany	Cross-sectional	50	Parkinson's disease	FEES	≥ 3	48.65%	61.54%	78.26%	29.63%
Järvenpää [36]	2021	Finland	Case–control	297	High risk group for dysphagia + healthy group	FEES	≥ 3	94.02%	96.11%	94.01%	96.11%

*VFSS* video fluoroscopic swallowing study, *FEES* fiberoptic endoscopic examination of swallowing, *PPV* positive predictive value, *NPV* negative predictive value

### Study Quality

**Fig. 2:**
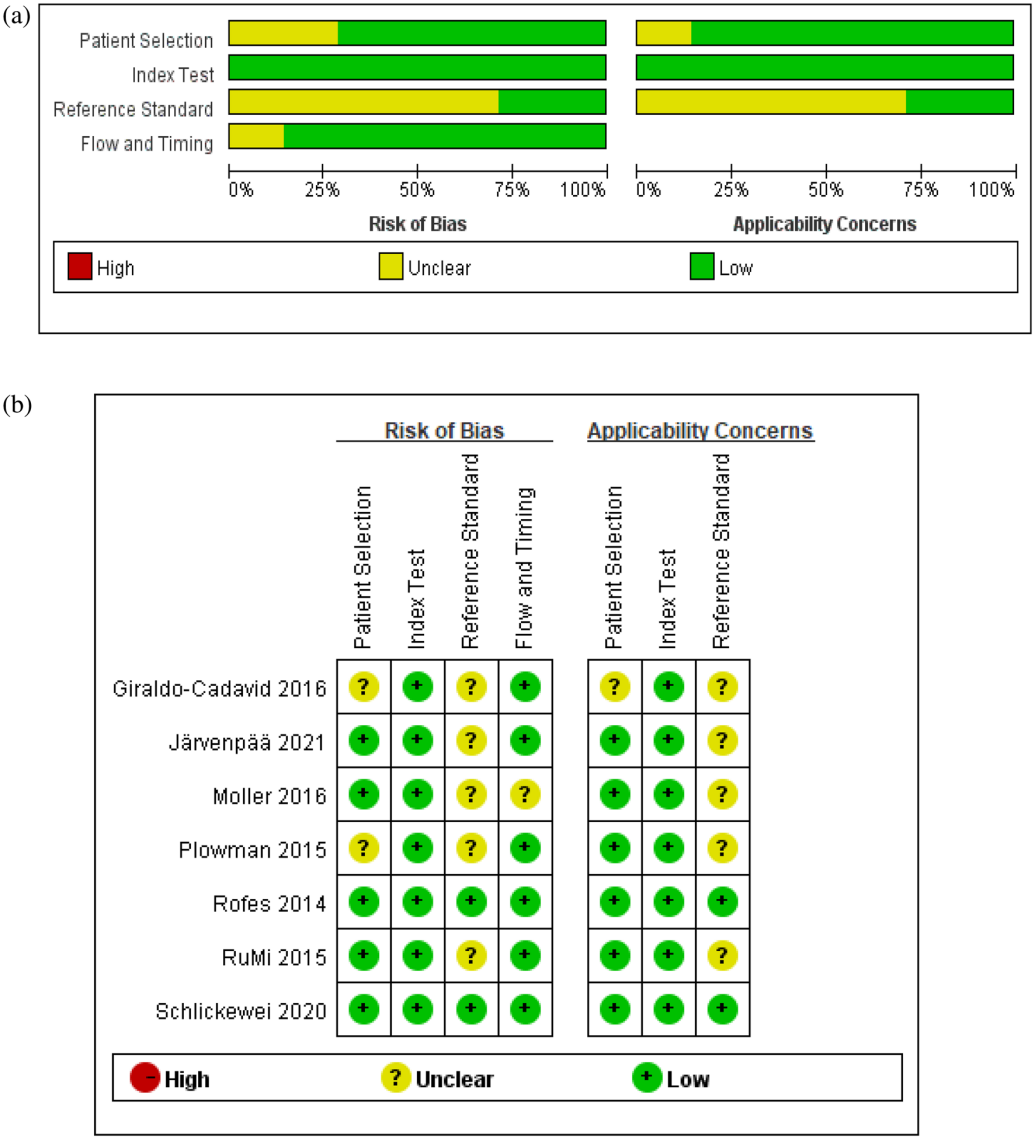
Assessment of the risk of bias for selected studies. **a** Risk of bias and applicability concerns graph. **b** Risk of bias and applicability concerns summary

Two researchers independently evaluated all included studies according to QUADAS-2 [54]. QUADAS-2 consists of 3 grades and 14 items (excluding the 3 non-essential items suggested by Cochrane). Among the seven studies, two were high quality and five were medium quality. A brief description of study quality is provided in Table 2. The results of the risk of bias assessment based on the Cochrane checklist are shown in Fig. 2. The study by Giraldo-Cadavid et al. [44] had an ambiguous risk with respect to patient selection and did not indicate whether patients were included consecutively. The studies by Schlickewei et al. [52] and Rofes et al. [49] were all blinded, but those by Järvenpää et al. [47], Rumi et al. [34], Plowman et al. [53], Möller et al. [36], and Giraldo-Cadavid et al. [44] did not explain whether blinding was used in the interpretation of the gold standard, and ambiguous attention was given to the gold standard. The reference standard for all studies correctly classified the target condition and used a PSA ≥ 2 to define dysphagia in advance. All studies avoided a case–control design.

**Table 2:** Quality assessment of studies

Study	Patient selection			Index test		Reference standard		Flow and timing				Quality grade
	Q1	Q2	Q3	Q4	Q5	Q6	Q7	Q8	Q9	Q10	Q11	
Rofes [39]	Yes	Yes	Yes	Yes	Yes	Yes	Yes	Yes	Yes	Yes	Yes	A
RuMi [23]	Yes	Yes	Yes	Yes	Yes	Yes	Unclear	Yes	Yes	Yes	Yes	B
Plowman [43]	Unclear	Yes	Yes	Yes	Yes	Yes	Unclear	Yes	Yes	Yes	Yes	B
Giraldo-Cadavid [33]	Unclear	Yes	Unclear	Yes	Yes	Yes	Unclear	Yes	Yes	Yes	Yes	B
Möller [25]	Yes	Yes	Yes	Yes	Yes	Yes	Unclear	Unclear	Unclear	Yes	Yes	B
Schlickewei [42]	Yes	Yes	Yes	Yes	Yes	Yes	Yes	Yes	Yes	Yes	Yes	A
Järvenpää [36]	Yes	Yes	Yes	Yes	Yes	Yes	Unclear	Yes	Yes	Yes	Yes	B

Q1: Could the selection of patients have introduced bias? Q2: Was a case–control design avoided? Q3: Did the study avoid inappropriate exclusions? Q4: Were the index test results interpreted without knowledge of the results of the reference standard? Q5: If a threshold was used, was it prespecified? Q6: Is the reference standard likely to correctly classify the target condition? Q7: Were the reference standard results interpreted without knowledge of the results of the index test? Q8: Was there an appropriate interval between index tests and reference standard? Q9: Did all patients receive a reference standard? Q10: did all patients receive the same reference standard? Q11: Were all patients included

### Meta-Analysis

#### Heterogeneity Test

When the EAT-10 cutoff values were 2 and 3, the Spearman’s correlation coefficients were 0.500 (*P* = 0.667) and 0.500 (*P* = 0.253), respectively, and there was no heterogeneity caused by the threshold effect. The *I*^2^ statistic was 12.5% (*P* = 0.32) using a cutoff value of 2, suggesting that there was no heterogeneity caused by a non-threshold effect, and it was 92.8% (*P* < 0.05) when using a cutoff value of 3. The heterogeneity of included studies was large, and a random-effects model was adopted.

#### Diagnostic Accuracy of EAT-10

**Fig. 3:**
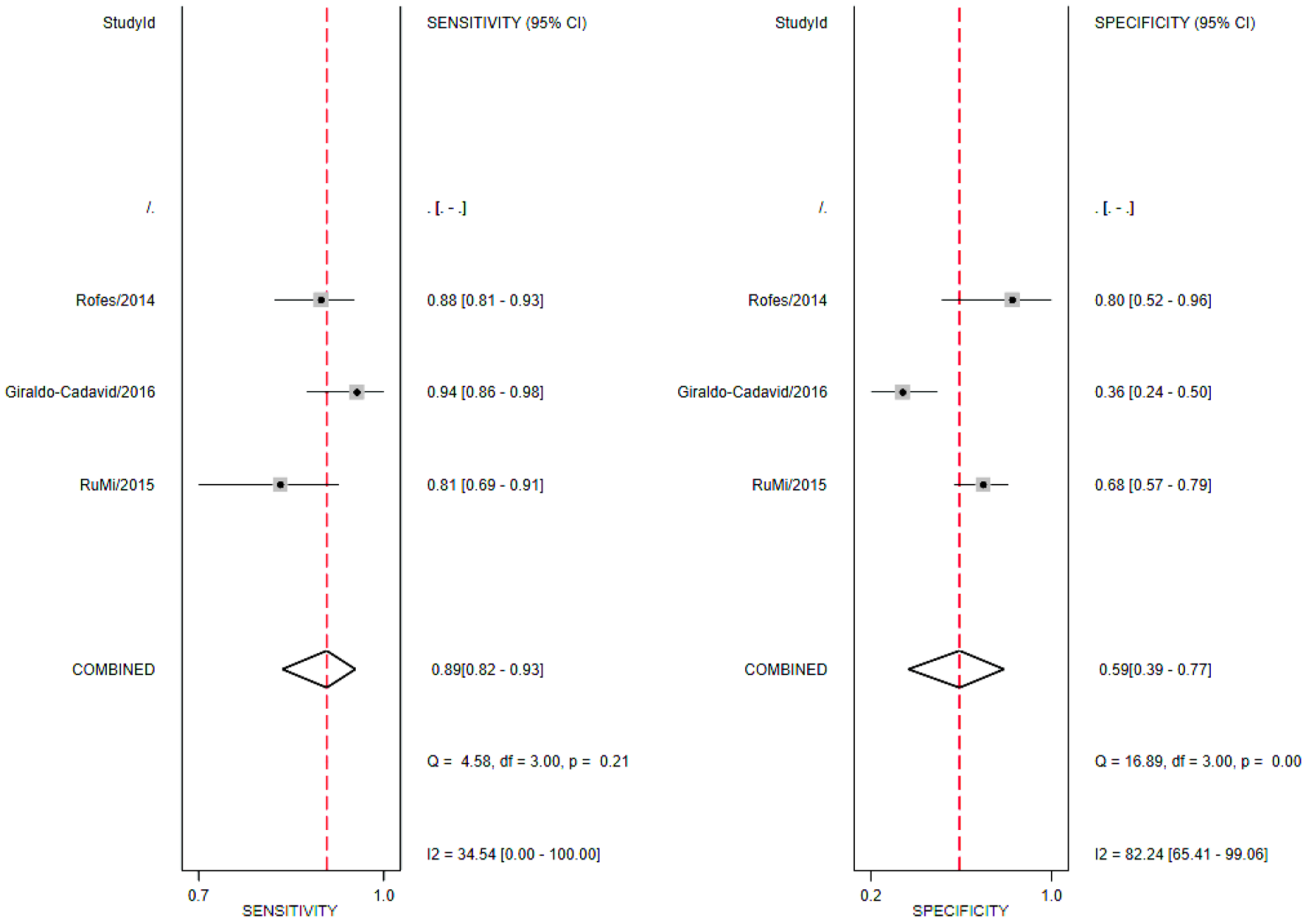
Forest plot for Eating Assessment Tool-10 at a cutoff value of 2

**Fig. 4:**
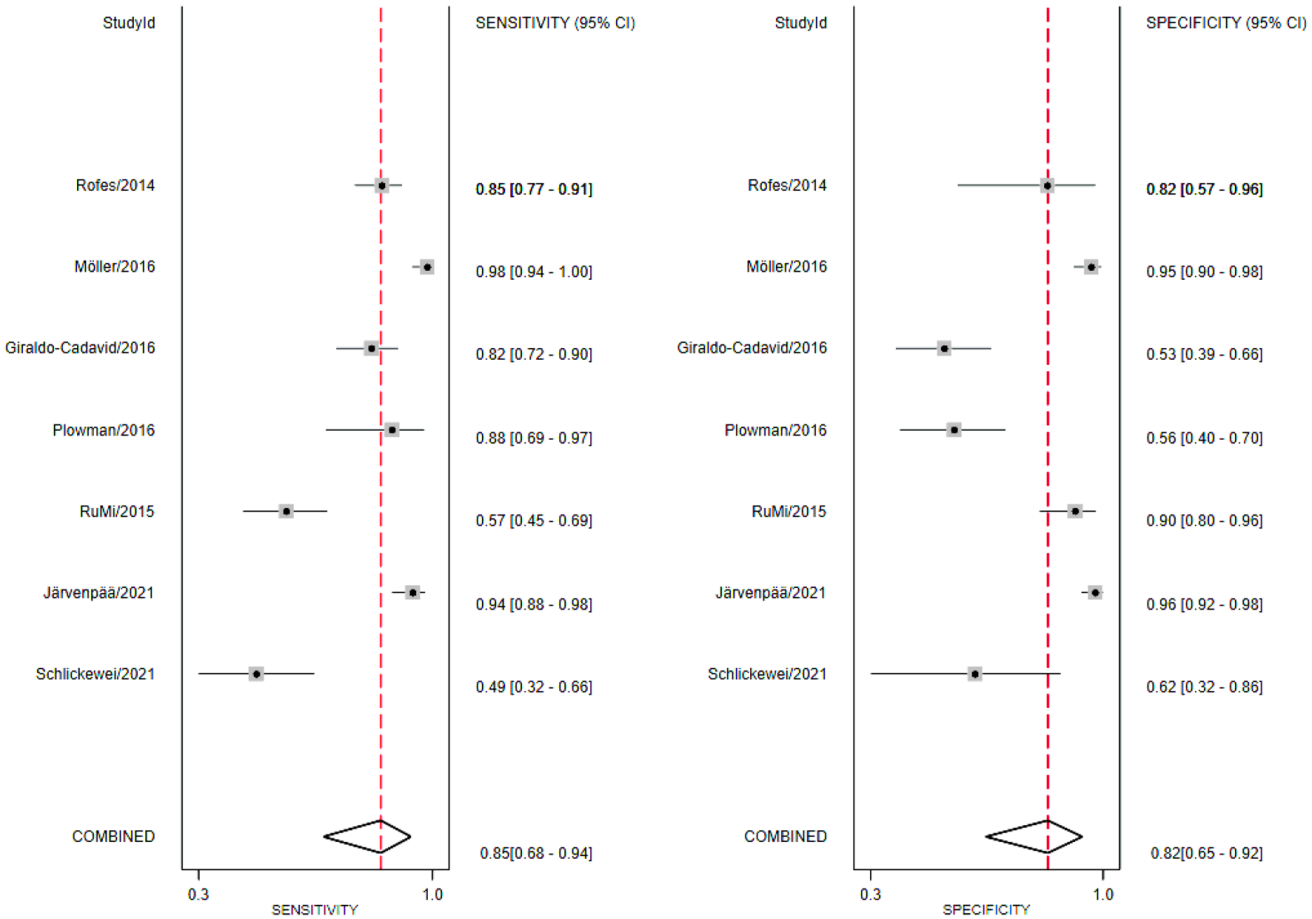
Forest plot for Eating Assessment Tool-10 at a cutoff value of 3

Using an EAT-10 cutoff value of 2, the pooled sensitivity, specificity, PLR, NLR, and DOR were 0.89 (95% CI 0.82–0.93), 0.59 (95% CI 0.39–0.77), 2.17 (95% CI 1.38–3.42), 0.19 (95% CI 0.13–0.29), and 11.49 (95% CI 5.86–22.53), respectively (Fig. 3). Using a cutoff value of 3, these values were 0.85 (95% CI 0.68–0.94), 0.82 (95% CI 0.65–0.92), 4.84 (95% CI 1.72–13.50), 0.18 (95% CI 0.07–0.46), and 26.24 (95% CI 5.06–135.95), respectively (Fig. 4). The diagnostic accuracy of EAT-10 at each cutoff value is described in Table 3.

**Table 3:** Result of meta-analysis

Study	Cutoff value	Sensitivity (95% CI)	Specificity (95% CI)	PLR (95% CI)	NLR (95% CI)	DOR (95% CI)
RuMi [23]	2	0.81 [0.69, 0.91]	0.68 [0.57, 0.79]	2.58 [1.81, 3.68]	0.27 [0.15, 0.48]	9.53 [4.12, 22.08]
Giraldo-Cadavid [33]		0.94 [0.86, 0.98]	0.36 [0.24, 0.50]	1.47 [1.19, 1.81]	0.18 [0.07, 0.44]	8.34 [2.89, 24.07]
Rofes [39]		0.88 [0.81, 0.93]	0.80 [0.52, 0.96]	4.40 [1.59, 12.12]	0.15 [0.09, 0.26]	29.14 [7.31, 116.19]
Summary	2	0.89 [0.82, 0.93]	0.59 [0.39, 077]	2.17 [1.38, 3.42]	0.19 [0.13, 0.29]	11.49 [5.86, 22.53]
Schlickewei	3	0.49 [0.32, 0.66]	0.62 [0.32, 0.86]	1.26 [0.59, 2.71]	0.83 [0.49, 1.42]	1.52 [0.42, 5.51]
Järvenpää [36]		0.94 [0.88, 0.98]	0.96 [0.92, 0.98]	24.18 [11.68, 50.05]	0.06 [0.03, 0.13]	388.37 [132.61, 1137.42]
RuMi [23]		0.57 [0.45, 0.69]	0.90 [0.80, 0.96]	5.93 [2.70, 13.03]	0.47 [0.35, 0.63]	12.55 [4.76, 33.09]
Plowman [43]		0.88 [0.66, 0.97]	0.56 [0.40, 0.70]	1.98 [1.39, 2.83]	0.22 [0.07, 0.64]	9.17 [2.40, 35.08]
Giraldo-Cadavid [33]		0.82 [0.72, 0.90]	0.53 [0.39, 0.66]	1.74 [1.29, 2.34]	0.34 [0.20, 0.58]	5.10 [2.33, 11.17]
Möller [25]		0.98 [0.94, 1.00]	0.95 [0.90, 0.98]	19.51 [9.47, 40.17]	0.02 [0.00, 0.07]	1056.00 [215.02, 5186.19]
Rofes [39]		0.85 [0.77, 0.91]	0.82 [0.57, 0.96]	4.82 [1.72, 13.5]	0.18 [0.11, 0.30]	26.63 [6.91, 102.64]
Summary	3	0.85 [0.68, 0.94]	0.82 [0.65, 0.92]	4.84 [1.72, 13.50]	0.18 [0.07, 0.46]	26.24 [5.06, 135.95]

*CI* confidence interval, *PLR* positive likelihood ratio, *NLR* negative likelihood ratio, *DOR* diagnostic odds ratio

**Fig. 5:**
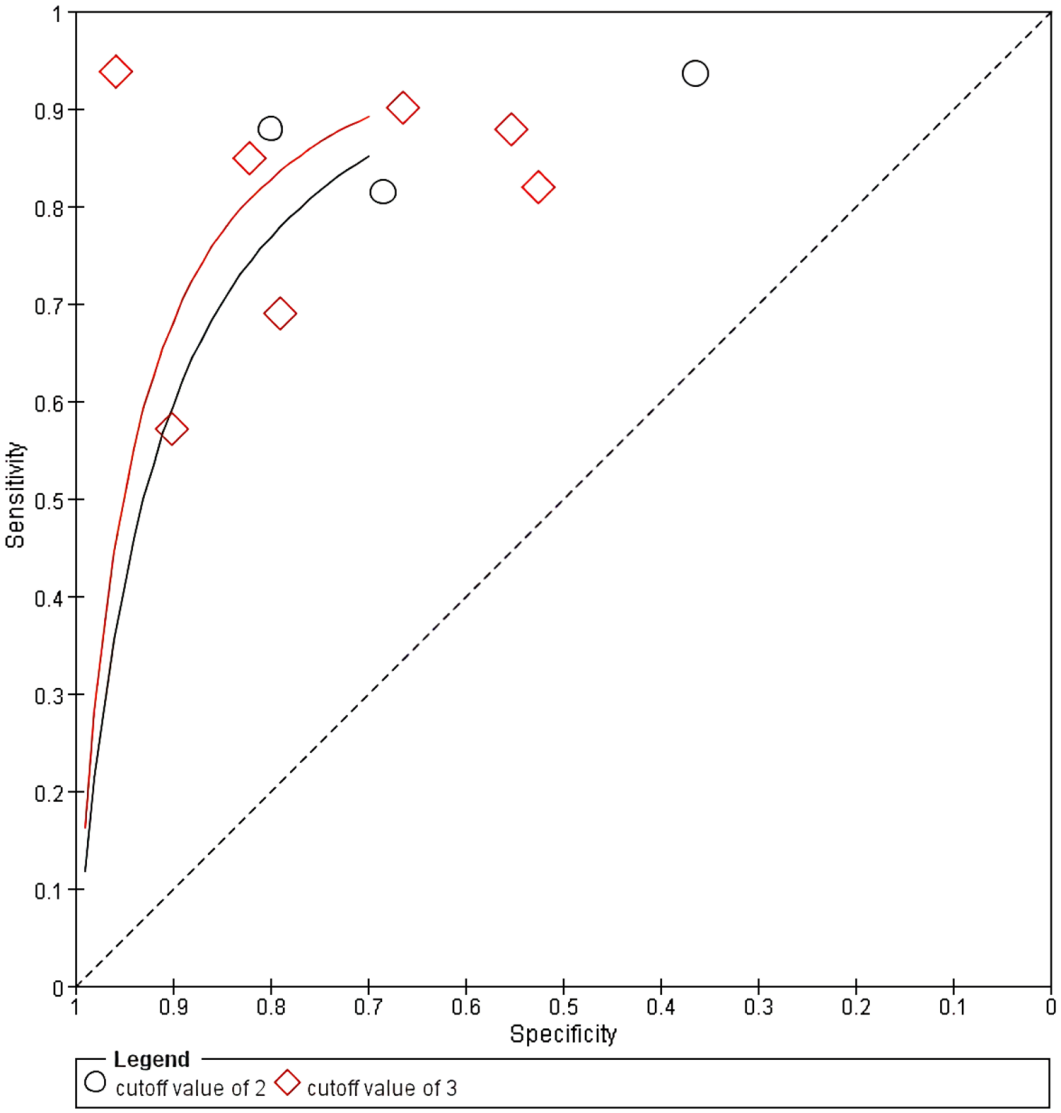
Summary receiver operating characteristic curves of Eating Assessment Tool-10 at cutoff values of 2 and 3

The SROC curves with cutoff values of 2 and 3 are plotted in Fig. 5, showing that the curve with a cutoff value of 3 is better than the one with a cutoff value of 2. Using a cutoff value of 2, the Q statistic was 0.80 and the AUC was 0.87 (95% CI 0.82–0.93). Using a cutoff value of 3, the Q statistic was 0.84 and the AUC was 0.90 (95% CI 0.88–0.93).

## Discussion

Dysphagia can cause serious complications, and early screening can help intervene in high-risk groups, effectively reducing dehydration, malnutrition, and aspiration pneumonia. However, even in hospitals, swallowing disorders are often ignored, dysphagia is often not reported by patients and ignored by clinicians, and the true prevalence of dysphagia may be higher than reported [55].

EAT-10 has been translated into various languages. The internal consistency between different versions (Cronbach’s *α*) ranges from 0.84 to 0.96 and ICCs range from 0.70 to 1.00. The internal consistency and retest reliability of the different EAT-10 versions are shown in Table 4.

**Table 4:** The internal consistency and intraclass correlation coefficients of the different version of the EAT-10

Version	Year	Anthor	Cronbach's *α*	ICC
Original English [11]	2008	Belafsky	0.96	0.72–0.91
Italian [41]	2013	Schindler	0.90	0.95
Chinese [38]	2015	Rumi	0.85	> 0.7
Portuguese [43]	2015	Nogueira	0.95	0.92–1.00
Hebrew [47]	2016	Abu-Ghanem	0.96	0.98
Spanish [48]	2016	Giraldo-Cadavid	0.91	0.94
Swedish [40]	2016	Möller	0.88	0.90
Turkish [49]	2016	Demir	0.90	0.91^a^
Pediatric [50]	2018	Serel Arslan	0.87	NM
Greek [45]	2018	Printza	0.96	NM
Hebrew [44]	2019	Shapira-Galitz	0.93	0.82^a^
French [46]	2019	Lechien	0.95	0.92^a^
Finland [51]	2021	Järvenpää	0.84	0.93

*ICC* intraclass correlation coefficients, *NM* not mentioned

^a^Retest reproducibility

EAT-10 has good predictive value for swallowing disorders caused by various diseases. EAT-10 scores in patients with head and neck cancer with dysphagia were significantly higher than those in patients without dysphagia (*P* < 0.05) [41]. When the EAT-10 cutoff value was 15, the sensitivity and specificity in predicting aspiration for nervous system diseases [56] was 81.0% and 58.0%, respectively. Using a cutoff value of 9, unilateral vocal fold paralysis aspiration [57] in patients was predicted with a sensitivity of 77.8% and specificity of 73.1%. EAT-10 scores can also predict the presence of aspiration in patients with ALS and chronic obstructive pulmonary disease (COPD). ALS [53] had a sensitivity of 86%, specificity of 76%, and a likelihood ratio of 3.1. Stable COPD [58] was observed in 91.67% and 77.78% of patients, and the DOR was 38.50. However, the ability of EAT-10 to predict penetration and aspiration in patients with PD was disappointing, with a sensitivity of only 58% at a cutoff of 6 [52]. This is because patients with PD usually have decreased laryngopharyngeal sensitivity, are unable to notice whether the residue after swallowing is removed, and the threshold of the cough reflex increases [59, 60], thus affecting the answers to questions 3–9. The pediatric version of EAT‐10 has a cutoff value of 4, with a sensitivity and specificity of 91.3% and 98.8%, respectively, which could effectively distinguish children with dysphagia from healthy children. EAT-10 has been found to correlate strongly (*r* = 0.41, *P* < 0.001) with the FEES PAS score [46].

Although many studies have reported the diagnostic accuracy of EAT-10, there is still no consensus on the best cutoff value for the diagnosis of dysphagia. Giraldo-Cadavid et al. [44] and Rofes et al. [49] believed that using 2 as the EAT-10 cutoff value had higher sensitivity and could avoid missing true diagnoses of any patient with dysphagia, but it could also increase the rate of misdiagnosis. Belafsky et al. [11], the authors of the original EAT-10, and many other researchers [36, 37, 39, 47, 53] suggested using 3 as the cutoff value because it has a better balance between sensitivity and specificity and a higher diagnostic accuracy. Giraldo-Cadavid et al. [44] also reported 94.3% sensitivity and 49.5% specificity when using 4 as an EAT-10 cutoff value. In this study, a meta-analysis of the most controversial values – 2 and 3 – was conducted to calculate their diagnostic performance and to determine the best cutoff value for EAT-10 in predicting patients with swallowing disorders.

There were 7 studies included, involving 1064 subjects from 7 different countries. Two studies were high quality and five studies were medium quality, with no significant risk deviation. Sensitivity and specificity are important indexes to evaluate screening tools. Sensitivity refers to the ability to screen out patients with illness or related symptoms and specificity is the ability to exclude patients without disease or related symptoms. The summary sensitivity of a cutoff of 2 was 4% higher than that of 3, but the specificity of a cutoff of 2 was 23% lower than that of 3 at the cost of greatly increasing the misdiagnosis rate. In this study, the DOR of the cutoff value of 3 is significantly higher than that of 2, indicating that using 3 as the EAT-10 cutoff value has a better discrimination effect for dysphagia. This screening tool should have high recognition ability for swallowing disorders. Although sensitivity is the primary indicator to consider for the screening tool, the diagnostic value of 3 is higher than that of 2 when specificity is taken into account. The AUC of the SROC curve reflects the accuracy of diagnostic tests. Our results show that EAT-10 has good accuracy for a cutoff value of 2 or 3, which can be applied in clinical screening of people with dysphagia. SROC curves consider both specificity and sensitivity. This study shows that an EAT-10 cutoff value of 3 has a diagnostic accuracy better than a cutoff of 2.

In this study, some factors affected the results of the sensitivity and specificity, which may arise from several factors. First, cultural differences can affect the clinical application of EAT-10. Shapira-Galitz et al. [27] found that the Hebrew version of EAT-10 was different in patients’ use of their mother tongue. The mean EAT-10 score of the dysphagia group was higher than that of English speakers, but lower than that of Italian, Spanish, or Swedish speakers and especially Japanese speakers. Even between the two Hebrew versions, there was a difference in the mean scores of the dysphagia group [43]. Languages that tend to score higher will show higher sensitivity and lower specificity in the EAT-10 diagnostic accuracy validation. Second, patients with obvious major psychiatric or cognitive disorders were excluded, as they would be unable to utilize the questionnaire. Mölle et al. [36] did not consider the cognitive status of their patients. As such, their study design does not accurately reflect the individual items in the EAT-10. Thus, when compared to the gold standard, the EAT-10 score results differ significantly from the gold standard, and the diagnostic accuracy of the resulting questionnaire is lower than the true values. Third, as penetration and aspiration are usually sporadic, VFSS and FEES may produce FN results and fail to identify the risk of penetration and aspiration in some high-risk groups [48, 61, 62], affecting EAT-10 sensitivity and specificity; in these studies, the sensitivity, specificity, and positive prediction values obtained were lower than true values. Fourth, the type of disease and the choice of the gold standard also affect the sensitivity and specificity of EAT-10. EAT-10 cannot accurately indicate patients with dysphagia caused by decreased sensitivity, such as those with PD [52]. The EAT-10 questionnaire completed by these patients may yield FN results, and so the sensitivity, positive predictive value, and negative predictive value obtained would be reduced compared to the true values. When patients with COPD are evaluated for swallowing disorders, FEES has better reliability than VFSS because it can better evaluate the pharyngeal mucosa, laryngopharyngeal sensitivity, and vocal cords. PAS scores in FEES are often higher than those with VFSS [62, 63]. Using VFSS as the gold standard, the sensitivity, specificity, and positive predictive value obtained would be somewhat lower compared to FEES.

EAT-10 also has good psychometric properties and reliability [11, 43–45, 64]; however, Cordier et al. [64] conducted Rasch analysis on EAT-10 and found that it has significant weaknesses in construction validity, including item redundancy, the lack of easy and difficult items, and different thresholds of rating scale categories. The second question (“My swallowing problem interferes with my ability to go out for meals”) is difficult to answer for some patients who have been hospitalized for a long time due to diseases and only present or are diagnosed with dysphagia during hospitalization. Item 2 may not be suitable for screening patients with dysphagia [34]. Therefore, EAT-10 needs to be improved for better clinical applications and research. (1) When designing the questionnaire, the questions should be designed from easy to difficult and from concrete to abstract. The EAT-10 can adjust the order of the items and consider putting items 2, 7, and 10 at the end of the questionnaire. (2) A Rasch analysis of the EAT-10 reveals that items 2, 7, and 10 do not contribute to the overall construct [64]. We should consider removing these three items from the EAT-10. (3) The second question (“My swallowing problem interferes with my ability to go out for meals”) is difficult to answer for some patients who have been hospitalized for a long time due to diseases and only present or are diagnosed with dysphagia during hospitalization. Item 2 may be unsuitable for screening patients with dysphagia [34]. “Go out’ focuses more on a patient’s mobility than on the impact of swallowing difficulties on life interactions. We can consider replacing item 2 with ‟My swallowing problem has affected my ability to eat with others.” (4) The Rasch analysis [64] also revealed that the EAT-10 was unable to form a second dimension that would allow further differentiation according to patient ability. Oral phase, esophageal phase, airway protection, and other related items can be considered in the EAT-10. Moreover, it is not suitable for swallowing disorders caused by decreased sensitivity [52].

## Conclusion

In summary, EAT-10 has been translated into many languages and is widely used in clinical practice. Although EAT-10 has some structural validity defects, it has good internal consistency, ICCs, and good psychometric properties. There was a linear correlation between EAT-10 and PAS scores, which could also reflect the severity of dysphagia to a certain extent. Using 2 and 3 as cutoff values showed good diagnostic performance. EAT-10 can be used as a preliminary screening tool for dysphagia. However, the diagnostic accuracy with a cutoff of 3 is higher, which can not only screen most high-risk groups of swallowing disorders but also avoid a high misdiagnosis rate. Therefore, a cutoff of 3 is recommended as the best cutoff value for EAT-10.
